# Enamel Matrix Derivative Enhances the Odontoblastic Differentiation of Dental Pulp Stem Cells via Activating MAPK Signaling Pathways

**DOI:** 10.1155/2022/2236250

**Published:** 2022-04-28

**Authors:** Beidi Zhang, Min Xiao, Xiaogang Cheng, Yu Bai, Hanze Chen, Qing Yu, Lihong Qiu

**Affiliations:** ^1^Department of Endodontics, School and Hospital of Stomatology, China Medical University, Liaoning Provincial Key Laboratory of Oral Diseases, Shenyang, China; ^2^Department of Stomatology, The First Affiliated Hospital of Soochow University, Suzhou, Jiangsu 215000, China; ^3^State Key Laboratory of Military Stomatology & National Clinical Research Center for Oral Diseases & Shaanxi Key Laboratory of Stomatology, Department of Operative Dentistry and Endodontics, School of Stomatology, The Fourth Military Medical University, Xi'an, China; ^4^Department of Neurology, Neuroscience Center, Sir Run Run Shaw Hospital, School of Medicine, Zhejiang University, Hangzhou, China

## Abstract

The odontoblastic differentiation of dental pulp stem cells (DPSCs) contributes to pulp-dentin regeneration. Enamel matrix derivative (EMD) is considered to be a critical epithelial signal to induce cell differentiation during odontogenesis and has been widely applied to clinical periodontal tissue regeneration. The purpose of this study was to explore the effect of EMD on DPSCs proliferation and odontoblastic differentiation, as well as the underlying mechanisms. We conducted *in vitro* and *in vivo* researches to get a comprehensive understanding of EMD. *In vitro* phase: cell proliferation was assessed by a cell counting kit-8 (CCK-8) assay; then, alkaline phosphatase (ALP) activity and staining, alizarin red staining, real-time RT-PCR, and western blot analysis were conducted to determine the odontoblastic potential and involvement of MAPK signaling pathways. *In vivo* phase: after ensuring the biocompatibility of VitroGel 3D-RGD via scanning electron microscopy (SEM), the hydrogel mixture was subcutaneously injected into nude mice followed by histological and immunohistochemical analyses. The results revealed that EMD did not interfere with DPSCs proliferation but promoted the odontoblastic differentiation of DPSCs *in vitro* and *in vivo*. Furthermore, blocking the MAPK pathways suppressed the EMD-enhanced differentiation of DPSCs. Finally, VitroGel 3D-RGD could well support the proliferation, differentiation, and regeneration of DPSCs. Overall, this study demonstrates that EMD enhances the odontoblastic differentiation of DPSCs through triggering MAPK signaling pathways. The findings provide a new insight into the mechanism by which EMD affects DPSCs differentiation and proposes EMD as a promising candidate for future stem cell therapy in endodontics.

## 1. Introduction

Dental pulp is the only vascularized connective tissue in teeth, which is surrounded by highly mineralized enamel, cementum, and dentin. It remains vulnerable to bacterial infections, dental trauma, or chemicals. These damages would eventually lead to pulpitis, pulp necrosis, and even periapical diseases. The current clinical routine treatment for necrotic or infected pulp tissue is root canal therapy (RCT), which has satisfactory clinical efficacy in controlling infection and eliminating pain. However, teeth that have undergone RCT are more susceptible to tooth fracture and discoloration due to loss of pulp tissue and consequent functional alterations [1–3]. The spring up and widespread application of tissue engineering technology makes it possible to achieve partial or complete pulp-dentin regeneration by biologically implanting stem cells, appropriate signaling factors and scaffolds into the prepared pulp cavity. Under the induction of biomolecules, the stem cells proliferate and differentiate into odontoblasts and regenerate dentin-pulp complex [3, 4].

Signaling molecules, especially epithelial signals, are key factors that determine the fate of dental stem cells during tooth development and physiological tissue regeneration procedures [5]. Enamel matrix proteins (EMPs) are a complex of proteins that synthesized and secreted by inner enamel epithelial cells from enamel organ and epithelial root sheath. During the development of the crown of teeth, EMPs were secreted to regulate the mineralization and maturation of enamel. When it comes to the root of the tooth, EMPs were excreted to induce nearby mesenchymal cells to differentiate into odontoblasts and cementoblasts. Therefore, EMPs are one of the most considerable epithelial signals that play a crucial role throughout the tooth development [6]. The components of EMPs are complex, including amelogenin, enamelin, ameloblastin, proteases, and multiple growth factors [7]. EMPs are generally extracted from porcine tooth germs, namely, enamel matrix derivative (EMD). Currently, EMD has been widely applied to clinical periodontal tissue regeneration and dental implantation [8, 9]. It is well documented that EMD could promote the proliferation and differentiation of periodontal ligament stem cells, mesenchymal stem cells (MSCs), and osteoblasts [10–12]. The application of EMD in periodontal tissue regeneration has gained mature experience, but its application in the field of endodontics is still in the initial and developing stage. So far, a few studies have explored its potential for enamel remineralization and direct pulp capping *in vitro* [13, 14]. A previous study evaluated the effect of a single concentration of EMD on the odontogenic differentiation of heterogeneous dental pulp cells (DPCs) *in vitro* [15]. Notably, DPCs are heterogeneous population represented by fibroblasts, neurons, endothelial cells, odonto-/osteoprogenitors, and inflammatory and immune cells [16]. Further sorting or purification is required to obtain largely pure dental pulp stem cells (DPSCs) with MSCs characteristics. However, whether EMD could promote DPSCs odontoblastic differentiation *in vitro* and *in vivo* and its underlying mechanisms remains unclear.

Nowadays, DPSCs have emerged as an outstanding cell source for endodontic regeneration due to its fantastic multilineage differentiation capability and abundant reserves. Especially, DPSCs are endowed with high proliferation, self-renewal, and the ability of odontogenic, angiogenic, and neurogenic differentiations to regenerate dental pulp containing multiple cell types [17, 18]. To realize the full potential of DPSCs, an ideal cell carrier is required to create a functional and optimized microenvironment that makes cells feel like at home [19]. Since traditional solid or moldable scaffolds could hardly accommodate the irregularity and curvature of the pulp cavity, the superiority of injectable cell carrier in endodontic regeneration has gradually displayed [20]. VitroGel 3D-RGD used in our study is a ready-to-use, xeno-free tunable hydrogel designed to imitate the endogenous physiological microenvironment for *in vitro* and *in vivo* 3D culture of cells. Besides, favorable interplay has been found between the hydrogel and a variety of cell types, including prostate cancer cells, breast cancer cells, and bone marrow stroma cells [21–23].

Mitogen-activated protein kinase (MAPK) signaling pathway is one of the vital mechanisms that take part in the regulation of cell proliferation and odonto/osteogenic differentiation [24, 25]. It has been reported that BMP-2, MTA, and Biodentine can promote DPSCs odontoblastic differentiation through triggering MAPK signaling pathways [26–29]. However, it is still uncertain whether the MAPK signaling pathways is involved in the EMD-induced odontoblastic differentiation of DPSCs.

In this work, we explored the influence of EMD on the odontoblastic differentiation and viability of DPSCs *in vitro*, and observed the potential of EMD in promoting DPSCs odontoblastic differentiation *in vivo* with VitroGel 3D-RGD served as an injectable scaffold. Then, we further investigated the potential role of MAPK signaling pathways during this procedure to provide theoretical foundations for the application of EMD in the biological treatment of pulpal and periapical diseases.

## 2. Materials and Methods

### 2.1. Isolation and Culture of DPSCs

Sound and complete third molars were collected, with written informed consent, from adults (18-25 years of age) at the Hospital of Stomatology of the Fourth Military Medical University. The study was approved by the Ethics Committee of the Fourth Military Medical University (Protocol Number: kq-2018018). The teeth were split along the cementoenamel junction under sterile condition. After being gently removed from the teeth, dental pulp tissue was minced and digested with 3 mg/mL collagenase type I (Sigma-Aldrich) at 37°C for 45 to 60 min. The digested mixtures were washed three times with alpha modification of Eagle's Medium (*α*-MEM, Gibco) supplemented with 10% fetal bovine serum (FBS, Gibco) and centrifuged at 1,000 rpm for 5 min. Subsequently, supernatants were removed and the cells were resuspended in *α*-MEM containing 20% FBS, 100 U/mL penicillin (Sigma-Aldrich), and 100 *μ*g/mL streptomycin (Sigma-Aldrich). The cells were then seeded in 6-well plates and incubated with 5% CO_2_ at 37°C. Once reaching 80% confluence, the cells were passaged to acquire single-cell cloning by serial dilution. Cells from passage 3 to 5 were used in this study, and all experiments were conducted more than three times.

### 2.2. Flow Cytometric Analysis

DPSCs at passage 3 were trypsinized and resuspended in phosphate buffered saline (PBS, Hyclone) containing 3% FBS at a density of 1 × 10^6^ cells/mL. Next, the cells were added to a microcentrifuge tube (100 *μ*L/tube) and incubated with mouse anti-human monoclonal antibodies (5 *μ*L/test; all purchased from BioLegend, USA), including CD29-PE (cat.no. 303004), CD34-PE (cat.no. 343506), CD45-PE (cat.no. 304008), CD90 (cat.no. 328110), CD105-PE (cat.no. 323206), CD146-PE (cat.no. 342004), and STRO-1-FITC (cat.no. 340105), in the dark for 1 h at room temperature. Cells incubated with IgG-PE were set as isotype control. Finally, the cells were resuspended in PBS with 3% FBS and assayed by flow cytometry (Becton & Dickinson).

### 2.3. Differentiation of DPSCs *In Vitro*

#### 2.3.1. Odonto/Osteogenic Differentiation

To induce odonto/osteogenic differentiation, DPSCs were cultured in *α*-MEM supplemented with 10%FBS, 10 mmol/L *β*-glycerophosphate, 50 mg/L ascorbic acid, 10 nmol/L dexamethasone, 100 U/mL penicillin, and 100 *μ*g/mL streptomycin (all purchased from Sigma-Aldrich). The odonto/osteogenic induction medium (OM) was replaced every 3 days. Following induction for 14 days, the cells were tested for differentiation ability by alizarin red staining.

#### 2.3.2. Adipogenic Differentiation

To induce adipogenic differentiation, DPSCs were cultured in *α*-MEM containing 10%FBS, 0.1 *μ*M dexamethasone, 0.2 mM indomethacin, 0.01 mg/mL insulin, 0.5 mM IBMX, 100 U/mL penicillin, and 100 *μ*g/mL streptomycin (all purchased from Sigma-Aldrich). The medium was changed every 3 days. After being induced for 21 days, the cells were tested for adipogenic differentiation ability by Oil red O staining.

### 2.4. Immunofluorescence Staining

DPSCs were fixed with 4% paraformaldehyde for 15 min and permeabilized with 0.1% Triton X-100 for 5 min. Then, the cells were blocked with 1% BSA in PBS for 30 min and incubated with anti-CD105 antibody (Santa Cruz, CA, USA), anti-CD146 antibody (Santa Cruz), and anti-STRO-1 antibody (R&D Systems Inc., USA) overnight at 4°C. After being incubated with appropriate secondary antibodies at 37°C for 1 h, the nuclei were stained with 4',6-Diamidino-2-phenylindole dihydrochloride (DAPI) for 15 min in dark place. The fluorescence images were detected by laser scanning confocal microscopy (Olympus, Japan).

### 2.5. EMD Preparation and Cell Viability

EMD gel (30 mg/mL and 0.7 mL) (Emdogain, Straumann, Basel, Switzerland) was diluted with a-MEM to the final concentration.

To determine the number of viable cells, a cell counting kit-8 (CCK-8, Dojindo, Japan) assay was conducted according to the technical manual. In brief, DPSCs were inoculated in 96-well plates at a density of 1 × 10^3^ cells/well and pre-incubated for 24 hours. Then, the medium was replaced by 100 *μ*L various concentrations of EMD (0, 25, 50, and 100 *μ*g/mL), with five repetitive samples for each concentration. At 1, 3, 5, 7, and 9 days after incubation, 10 *μ*L of the CCK-8 solution was added to each well. After being incubated at 37°C for 3 hours, the absorbance was measured at 450 nm in a microplate reader (Power Wave 340, Bio-TEK, USA).

### 2.6. Alkaline Phosphatase (ALP) Activity and Staining

DPSCs were seeded at a density of 2 × 10^5^ cells/well into 6-well plates. When the cells reached 80% confluency, they were randomly divided into five groups: a control group, in which the cells were maintained in normal culture medium; an OM group, in which the cells were cultured in OM without EMD; and three EMD groups, in which different concentrations of EMD (25, 50, and 100 *μ*g/mL) were added to the OM group. At 3, 7, and 14 days after incubation, ALP content was evaluated using the Alkaline Phosphatase Assay Kit (Beyotime, China).

Cells were seeded at a density of 1 × 10^5^ cells/well into 12-well plates and cultured in OM with or without EMD. After 7 days of culture, the cells were stained with the BCIP/NBT Alkaline Phosphatase Color Development Kit (Beyotime) in accordance with the manufacturer's protocol.

### 2.7. Alizarin Red Staining and Quantification

Cells were seeded into 12-well plates and cultured in OM with or without EMD. After 14 days of incubation, cells were fixed with 4% paraformaldehyde for 30 min. Then, cells were stained with Alizarin red S (pH 4.2) for 5 min and photographed for mineralized nodules.

To quantify the alizarin red staining, 1 mL of 10% cetylpyridinium chloride was added to each well to dissolve the mineralized nodules. After 30 min, the dissolved solution was transferred into a 96-well plate and measured the absorbance at 562 nm.

### 2.8. Real-Time Reverse Transcription-Polymerase Chain Reaction (Real-Time RT-PCR)

DPSCs at days 3, 7, and 14 were collected and extracted for total cellular RNA with TRIzol reagent (Takara Bio Inc., Japan). Then, the reverse transcription reaction was proceeded using PrimeScript™ RT reagent Kit with gDNA Eraser (Takara Bio Inc., Japan), following the product manual of the manufacturer. Real-time RT-PCR was performed in an Applied Biosystems 7500 Real-Time PCR System (Applied Biosystems, Foster City, CA, USA) with TB Green® Premix Ex Taq™ II (Tli RNaseH Plus, Takara Bio Inc., Japan). And the relative expression levels of target genes were normalized to the expression of GAPDH using the 2^-*ΔΔ*Ct^ method. Primer sequences of target genes are detailed in Table 1.

### 2.9. Western Blot Analysis

DPSCs were collected to evaluate the odontoblastic differentiation after EMD treatment at days 3, 7, and 14. And cells induced with EMD for 0, 5, 10, 30, 60, and 120 min were collected to investigate the involvement of MAPK signaling pathways. After being serum starved for 24 h, DPSCs were blocked with specific signaling pathway inhibitors (SP600125 targeting JNK, SB203580 targeting p38 MAPK, and U0126 targeting ERK). Total proteins were quantified using a bicinchoninic acid protein assay kit (Beyotime). Proteins were separated on 10% TGX Stain-Free polyacrylamide gels (Bio-Rad, USA) and then transferred to Immun-Blot polyvinylidene difluoride membranes (Bio-Rad). Primary antibodies used were runt-related transcription factor 2 (RUNX2) (12556, Cell Signaling Technology, CST), ALP (ab108337, Abcam), bone sialoprotein (BSP) (5468, CST), dentin matrix protein 1 (DMP1) (ab103203, Abcam), dentin sialophosphoprotein (DSPP) (sc-73632, Santa Cruz), osteocalcin (OCN) (sc-390877, Santa Cruz), JNK (9252, CST), p-JNK (4668, CST), p38 MAPK (8690, CST), p-p38 MAPK (4511, CST), ERK1/2 (4695, CST), p-ERK1/2 (4370, CST), and glyceraldehyde-3-phosphate dehydrogenase (GAPDH) (60004-1-lg, Proteintech). ChemiDoc™ MP imaging system (Bio-Rad) and Image Lab™ software (Bio-Rad) were applied for digital imaging and analysis.

### 2.10. VitroGel 3D-RGD Hydrogel Preparation

#### 2.10.1. 3D Cell Culture

DPSCs were trypsinized and resuspended in *α*-MEM containing 30% FBS at a density of 1 × 10^6^ cells/mL. Then, the VitroGel 3D-RGD solution (TheWell Bioscience, USA) was diluted with VitroGel Dilution Solution (TheWell Bioscience) at 1 : 3 v/v ratio and gently mixed with cell suspension at 2 : 1 v/v ratio. Afterwards, the cell-hydrogel mixture was transferred to a well plate and left at room temperature for 20 min to form a soft gel. Finally, additional cell culture medium was added to each well to further stabilize the hydrogel.

#### 2.10.2. Scanning Electron Microscopy (SEM)

Cells were seeded into 24-well plates at a density of 1 × 10^5^ cells per well with 300 *μ*L hydrogel mixture. Hydrogel without cells served as the control group. After 3 and 7 days of incubation, the hydrogel was fixed with 2.5% glutaraldehyde at 4°C, frozen at -80°C overnight, and dried in a freeze-dryer (FD5–2.5 SIM) for about 24 h. Samples were sputtered with gold and detected by a scanning electron microscope (HitachiS3400 N, Japan).

### 2.11. Subcutaneous Injection *In Vivo*

To further investigate the effect of EMD on the odontoblastic differentiation of DPSCs *in vivo*, we conducted animal experiments in female immunodeficient mice (6 weeks old, BALB/c nude mouse, Beijing Vital River Laboratory Animal Technology Co. Ltd). All animal procedures were approved by the Laboratory Animal Care and Welfare Committee, School of Stomatology, Fourth Military Medical University. The cell-hydrogel mixtures were prepared the same as previously described for 3D cell culture. The mixtures combined with EMD were the experimental group, and those cells alone were the control group. Nude mice were anesthetized by intraperitoneal injection of 1% pentobarbital sodium (50 mg/kg). And then 600 *μ*L mixture of each group was subcutaneously injected into the dorsa of nude mice in random order. Each nude mouse possessed two injection sites. Mice were sacrificed six weeks after injection. All samples were retrieved and immediately fixed with 4% paraformaldehyde for 24 h at 4°C, and then processed for histological analyses.

### 2.12. Histological and Immunohistochemical Analyses

Harvested samples were decalcified with 10% EDTA (pH 7.4) and embedded in paraffin, and then sectioned for hematoxylin–eosin (H&E) staining and Masson's trichrome staining. Primary antibodies used for immunohistological analyses were rabbit anti-human DSPP polyclonal antibody (1 : 100, ab216892, Abcam) and mouse anti­human OCN monoclonal antibody (1 : 100, MAB1419, R&D Systems). After incubated with corresponding anti-rabbit or anti-mouse biotinylated secondary antibodies, the specimens were stained with DAB chromogen solution and counterstained with hematoxylin, then finally mounted for observation.

### 2.13. Statistical Analysis

Data are expressed as means ± standard deviation (SD) of triplicate independent measurements and analyzed by SPSS 20.0 software. One-way ANOVA or Student's *t* test was used to evaluate the significant difference. *P* < .05 was determined statistically significant.

## 3. Results

### 3.1. Isolation and Characterization of Human DPSCs

**Figure 1:**
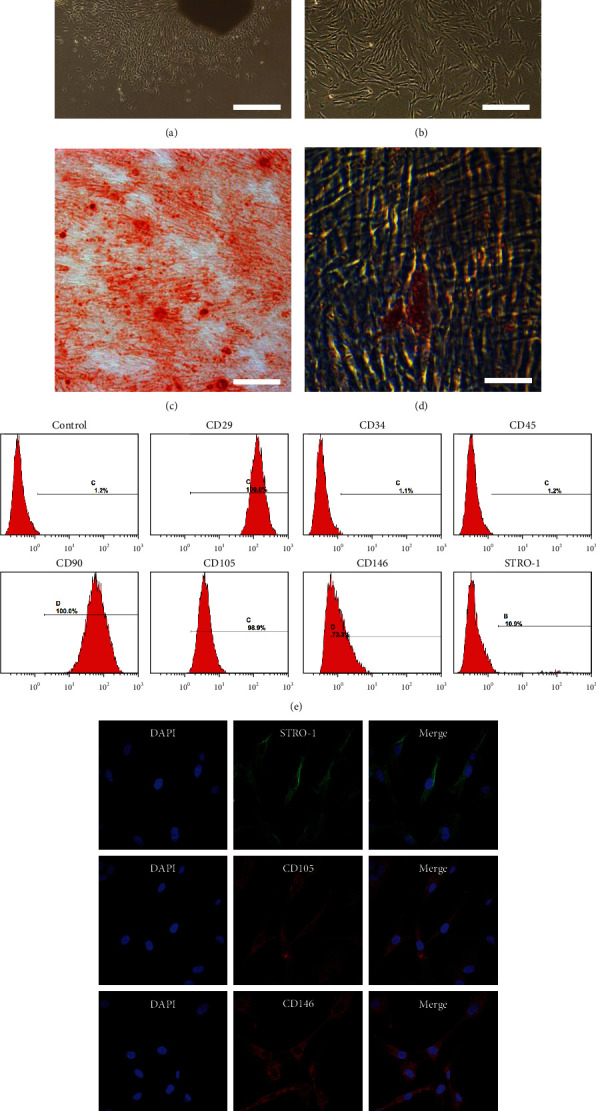
Culture and identification of dental pulp stem cells (DPSCs). (a) Morphology of primary DPSCs at day 5. (b) Colony-forming DPSCs obtained by limiting dilution assay. (c) Alizarin red staining of cells after odonto/osteogenic induction for 14 days. (d) Oil red O staining of cells after adipogenic induction for 21 days. (e) Flow cytometric analysis demonstrated that cells were negative for CD34 and CD45, but positive for CD29, CD90, CD105, CD146, and STRO-1. Cells incubated with IgG-PE were served as isotype control. (f) Immunofluorescence staining displayed positive expression of mesenchymal stem cells surface antigens STRO-1, CD105, and CD146. Nuclei were counterstained with DAPI. Scale bar =200 *μ*m (a, c); 100 *μ*m (b, d); 50 *μ*m (f).

Attachment of primary cells to the well plates was observed normally on the fifth day after their isolation from dental pulp tissue, and the primary cell clusters displayed spindle or star-like growth (Figure 1(a)). Single-cell clones were obtained by limiting dilution assay, and the cells within each colony appeared a representative fibroblast-like morphology (Figure 1(b)). The results of flow cytometric analysis revealed that these cells were negative for CD34 and CD45, but positively expressed CD29, CD90, CD105, CD146, and STRO-1, with a positive cell rate of 100%, 100%, 98.9%, 73.3%, and 10.9%, respectively (Figure 1(e)). Multilineage differentiation testing indicated that the obtained cells were successfully induced towards osteogenic and adipogenic lineages (Figures 1(c) and 1(d)). Immunofluorescence staining also displayed positive expression of MSCs surface antigens STRO-1, CD105, and CD146 (Figure 1(f)). The aforesaid tests displayed that these expanded cells possessed MSCs properties, and human DPSCs were successfully isolated from sound dental pulp tissue.

### 3.2. Effects of EMD on the Proliferation and Odontoblastic Differentiation of DPSCs *In Vitro*

**Figure 2:**
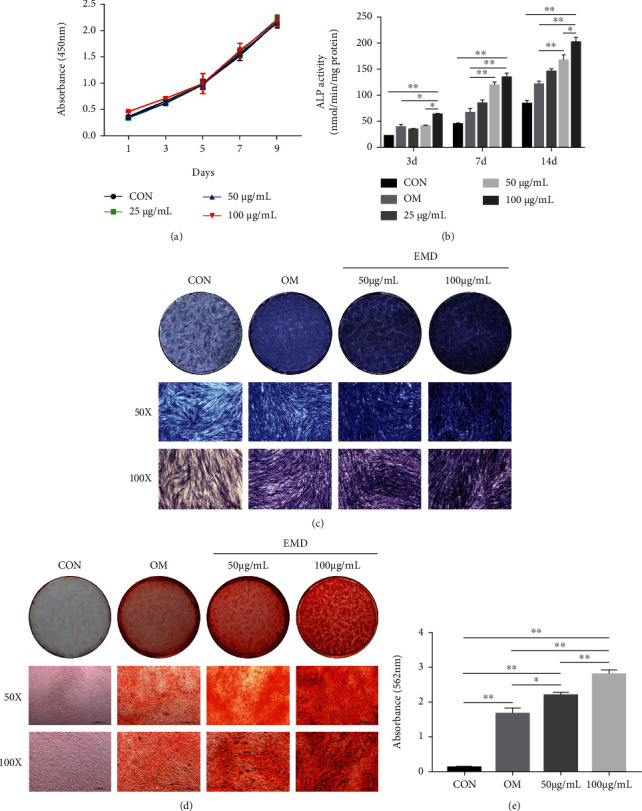
Screening for the optimal concentration of EMD and investigating the proliferation of EMD-treated DPSCs. (a) The effect of EMD on DPSCs proliferation was detected by a cell counting kit-8 assay on days 1, 3, 5, 7, and 9. (b) The ALP activity of DPSCs treated with various concentrations of EMD for 3, 7, and 14 days, respectively. (c) ALP staining of DPSCs treated with 50 and 100 *μ*g/mL of EMD at day 7. (d) and (e) Alizarin red staining and quantification of EMD-treated DPSCs at day 14. The results are presented as means ± SD of three independent experiments (*n* =3). ∗*P* < 0.05, ∗∗*P* < 0.01.

The results of CCK-8 showed that each concentration of EMD had little effect on DPSCs proliferation, and no significant differences were observed between the EMD groups and the control group among all tested time points (Figure 2(a)).

**Figure 3:**
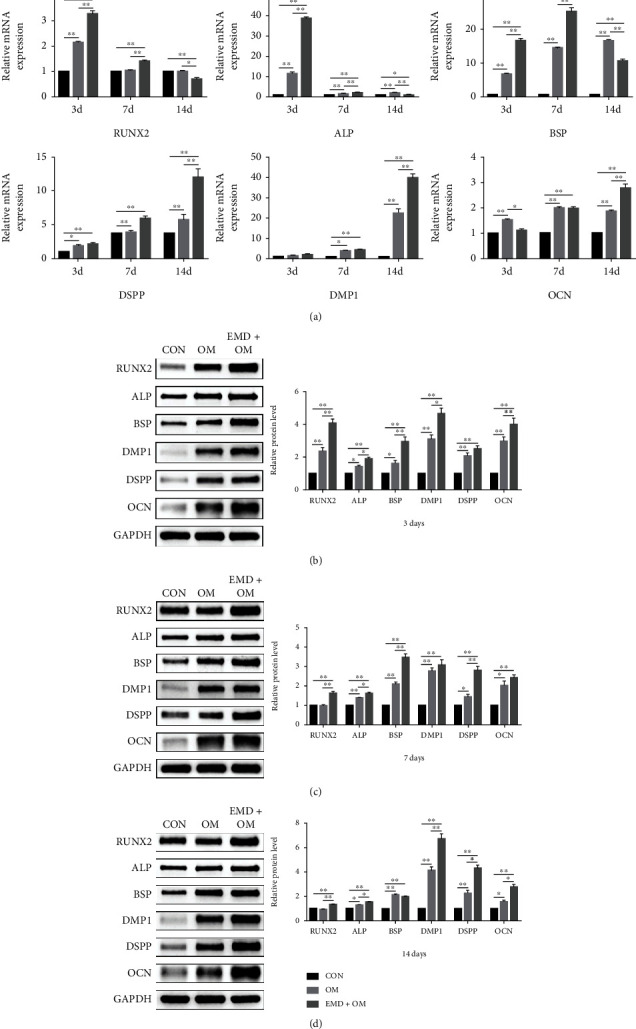
The influence of EMD on odontoblastic differentiation of DPSCs *in vitro*. (a) The expression levels of odonto/osteogenic-related marker genes (RUNX2, ALP, BSP, DSPP, DMP1, and OCN) were detected by real-time RT-PCR at days 3, 7, and 14. (b)–(d) Relative protein expression levels of odonto/osteogenic-related markers were detected by western blot analysis at days 3, 7, and 14. The results were quantified using Image Lab™ software. All data are displayed as means ± SD by three individual tests (*n* =3). ∗*P* < 0.05, ∗∗*P* < 0.01.

Then, ALP activity and staining, alizarin red staining, real-time RT-PCR, and western blot analysis were conducted to determine the optimal concentration of EMD and its influence on DPSCs differentiation. The ALP activity increased over time among all EMD-treated groups in general (Figure 2(b)). At days 7 and 14, the ALP content of the 50 and 100 *μ*g/mL group was markedly higher than the OM and control group. Compared with other groups, the 100 *μ*g/mL group significantly increased ALP activity at all tested time points. The results of ALP staining further confirmed the same conclusion (Figure 2(c)). Similarly, there was more calcium deposition in the 100 *μ*g/mL group than in the other groups at day 14 (Figures 2(d) and 2(e)). Therefore, we chose 100 *μ*g/mL as the optimum concentration of EMD for subsequent analyses. The real-time RT-PCR results revealed that EMD upregulated the expression levels of odonto/osteogenic-related marker genes in the early or late stages of mineralization (Figure 3(a)). Specifically, the mRNA expression levels of RUNX2, ALP, and BSP in the EMD+OM group were significantly higher than the control and OM group at day 3, while the odontoblastic-related genes including DSPP, DMP1, and OCN were remarkably upregulated at day 14. Furthermore, the protein levels of these genes changed in consistent with their mRNA expression levels (Figures 3(b)–3(d)). Compared with the other groups, the protein expression levels of RUNX2 and ALP in the EMD+OM group were significantly higher at all tested time points. The protein expression level of BSP in the EMD+OM group was dramatically higher than that in the control and OM group at days 3 and 7, while the odontoblastic-related genes DSPP, DMP1, and OCN were remarkably upregulated mainly in the late stage of mineralization.

### 3.3. Effects of EMD on DPSCs Odontoblastic Differentiation and Regeneration *In Vivo*

**Figure 4:**
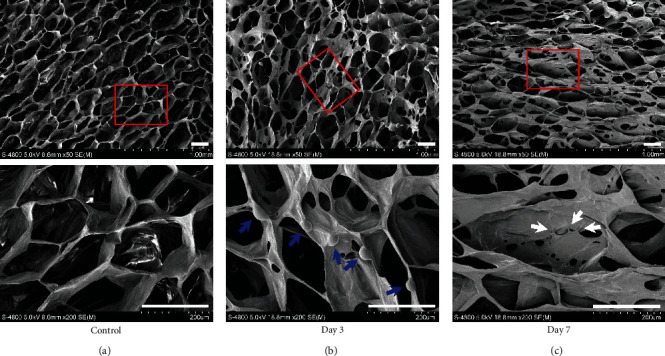
The ultrastructural feature of VitroGel 3D-RGD and its effect on the growth of DPSCs. (a) The surface ultrastructure of the hydrogel was detected via scanning electron microscopy (SEM). (b) and (c) SEM imaging of DPSCs cultured in the hydrogel system at day 3 and 7, respectively. Red boxed areas in the first row were zoomed in and displayed in the second one. The cells attached near the pore wall were pointed out with blue arrows. White arrows indicate cellular pseudopodia. The images were captured from three independent tests (*n* =3). Scale bar =200 *μ*m.

**Figure 5:**
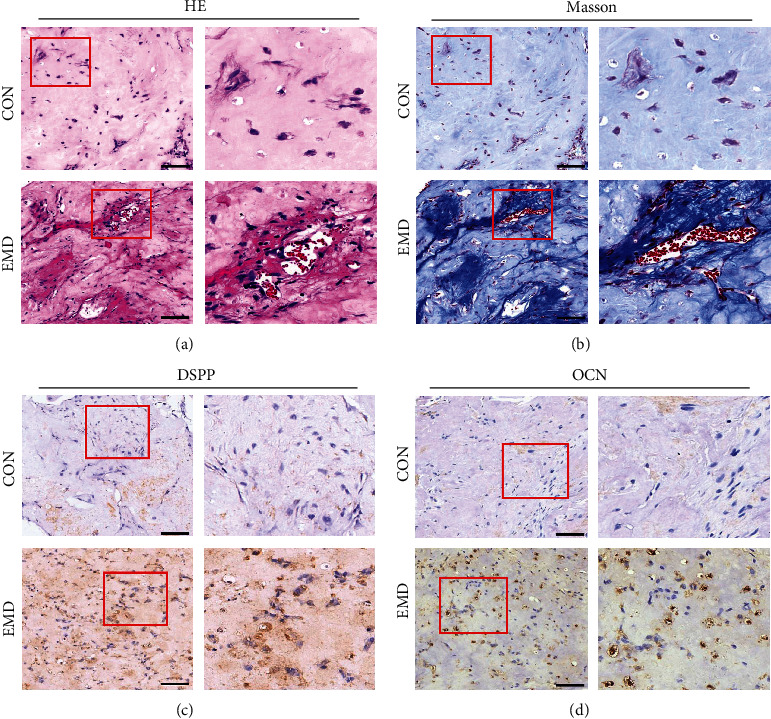
The effect of EMD on DPSCs odontoblastic differentiation and regeneration *in vivo*. Histological and immunohistochemical analyses of tissue regenerated after ectopic subcutaneous injection for six weeks (*n* =8). (a) H&E staining for collagen fibrils (light pink areas) and erythrocytes (bright red). (b) Masson's trichrome staining for collagen matrix (light blue areas), osteoid dentin (dark blue areas), and erythrocytes (bright red). (c) and (d) Immunohistochemical staining of DSPP and OCN, respectively. The dark brown areas in the extracellular matrix indicated positive expression of DSPP and OCN. Red boxed areas were zoomed in and presented on the right side of the corresponding image. Scale bar =100 *μ*m.

We first observed the growth of DPSCs cultured in the VitroGel 3D-RGD system to verify whether the hydrogel has good biocompatibility with DPSCs. SEM imaging of the control group showed that there was plenty of uniform and interconnected pores in the hydrogel (Figure 4(a)). The cells at day 3 were evenly distributed near the pore wall like semicircular spheres, with some extracellular matrix was secreted (Figure 4(b)). After 7 days of cultivation, the pore structure of hydrogel gradually disappeared and replaced by abundant extracellular matrix and cellular pseudopods (Figure 4(c)). Due to its high porosity and good biocompatibility with DPSCs, VitroGel 3D-RGD is suitable for *in vivo* studies as an injectable hydrogel [30]. Then, we conducted *in vivo* experiment by ectopic subcutaneous injection. After 6 weeks of operation, the specimens could easily distinguish from surrounding tissue and then sectioned for histological and immunohistochemical analyses. H&E staining illustrated that more collagen matrix and mineralized tissue were formed in the EMD group compared to the control group (Figure 5(a)). Similarly, Masson's trichrome staining found that the EMD group had abundant dentin matrix deposition and more newly formed blood vessels compared with the control group (Figure 5(b)). Besides, the cells exposed to EMD displayed stronger positive expression of DSPP and OCN, as detected by immunohistochemical staining (Figures 5(c) and 5(d)). In general, these results illuminated that EMD has the capacity for promoting the odontoblastic differentiation of DPSCs *in vivo*.

### 3.4. Involvement of the MAPK Signaling Pathways in the EMD-Enhanced Odontoblastic Differentiation of DPSCs

**Figure 6:**
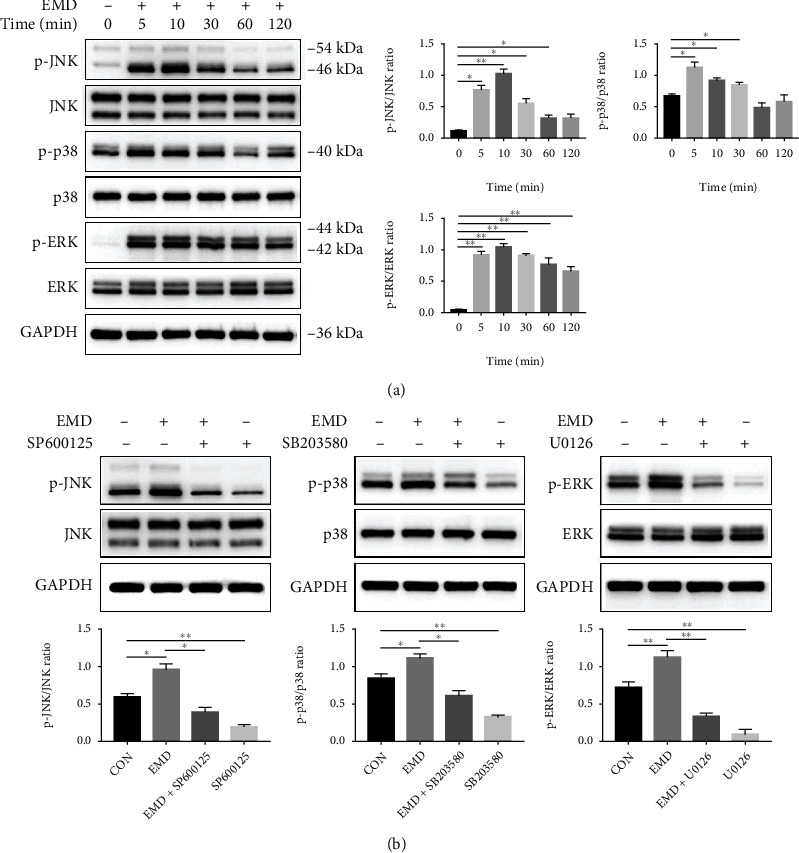
EMD activated the MAPK signaling pathways in DPSCs. (a) Protein expression levels of JNK, p-JNK, p38 MAPK, p-p38 MAPK, ERK1/2, and p-ERK1/2 in EMD-treated DPSCs were detected by western blot analysis at different time points (0, 5, 10, 30, 60, and 120 min). (b) DPSCs were pretreated with specific pathway inhibitors (SP600125 targeting JNK, SB203580 targeting p38 MAPK, and U0126 targeting ERK) for 1 h and then stimulated with EMD. SP600125 and U0126 group were stimulated for 10 min, and SB203580 group was stimulated for 5 min. The effectiveness of the inhibitors was verified by western blot assay. Quantification was done by Image Lab™ software. The results are presented as means ± SD of three independent experiments (*n* =3). ∗*P* < 0.05, ∗∗*P* < 0.01.

To investigate the mechanism of EMD-enhanced differentiation of DPSCs, protein levels of JNK, p-JNK, p38 MAPK, p-p38 MAPK, ERK1/2, and p-ERK1/2 were detected by western blot analysis. The results showed that the protein level of p-JNK and p-ERK1/2 increased rapidly after 5 min, peaked at 10 min, and then declined gradually (Figure 6(a)). In addition, the protein level of p-p38 MAPK increased immediately and reached the highest level at 5 min. The ratios of p-JNK/JNK, p-p38 MAPK/p38 MAPK, and p-ERK/ERK further revealed that EMD activated the JNK, p38 MAPK, and ERK pathways in DPSCs.

**Figure 7:**
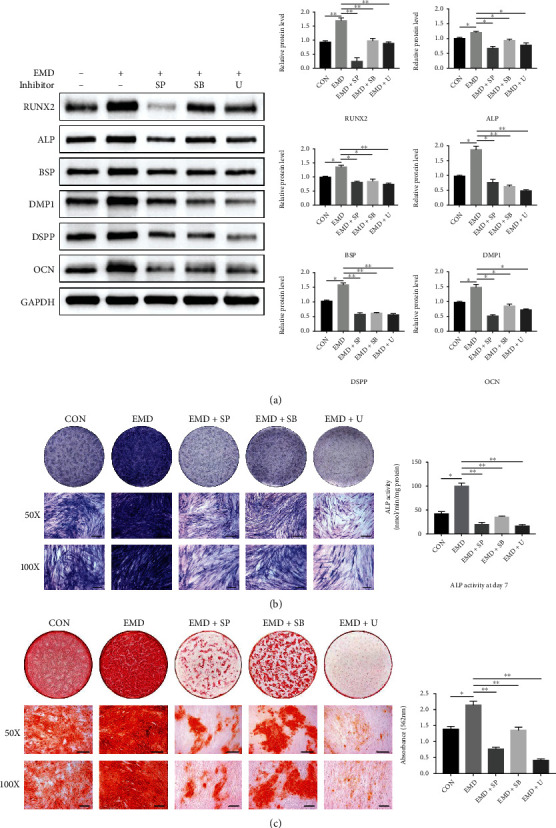
Blocking the MAPK signaling pathways inhibited odontoblastic differentiation of EMD-treated DPSCs. Cells were divided into five group: EMD, EMD+SP600125, EMD+SB203580, EMD+U0126, and a control group which cultured in OM alone. (a) Protein levels of RUNX2, ALP, BSP, DMP1, DSPP, and OCN were measured by western blot analysis at day 7. Quantification was done by Image Lab™ software. (b) ALP staining and ALP activity assay at day 7. (c) Alizarin red staining and quantification at day 14. All data are presented as means ± SD of three independent experiments (*n* =3). ∗*P* < 0.05, ∗∗*P* < 0.01.

To further explore the involvement of MAPK signaling pathways during the EMD- induced differentiation of DPSCs, specific pathway inhibitors SP600125, SB203580, and U0126 were applied for blocking the JNK, p38 MAPK, and ERK pathways, respectively. The effectiveness of the inhibitors was verified by western blot assay. Compared with the EMD group, the protein levels of p-JNK, p-p38 MAPK, and p-ERK1/2 were significantly suppressed by their corresponding inhibitors (Figure 6(b)). Then, we conducted western blot, ALP activity and staining, and alizarin red staining after blocking off the MAPK pathways. DPSCs were divided into five groups: a control group, in which the cells were cultured in OM alone; an EMD-treated group, in which 100 *μ*g/mL of EMD was added to the control group; and three EMD+ inhibitor groups, in which three specific MAPK pathway inhibitors were added to the EMD group. The protein levels of RUNX2, ALP, BSP, DMP1, DSPP, and OCN were dramatically decreased in various degrees after being blocked with inhibitors (Figure 7(a)). In addition, the ALP activity and mineralized nodules deposition, which were enhanced by EMD, were remarkably suppressed in the three EMD+ inhibitor groups (Figures 7(b) and 7(c)). Therefore, blocking the MAPK pathways inhibited odontoblastic differentiation of EMD-treated DPSCs.

## 4. Discussion

The desire for seeking biological alternatives to root canal treatment has spurred regenerative endodontics, which is biologically based procedure designed to physiologically replace damaged tooth structures [31]. Dental pulp and dentin are both histologically derived from the dental papilla of tooth germs. They are biological as a whole, namely, dentin-pulp complex, which response connectively to the external stimulus. Successful pulp-dentin regeneration relies profoundly on the odontoblastic differentiation of stem cells [32]. And the epithelial signals in the dental microenvironment are of tremendous significance to induce odontogenic differentiation [33]. Since EMD is the crucial epithelial signal that activates and regulates odontoblastic differentiation during tooth development, we hypothesized that EMD might be able to promote DPSCs odontoblastic differentiation by formulating a favorable microenvironment [34]. The EMD gel (Emdogain®, Straumann) used in our study is a commercial product that guarantees the activity and stability of the protein. Although EMD has been used clinically in the treatment of periodontal diseases with periodontal hard and soft tissue lost, less is known about its function in promoting DPSCs odontoblastic differentiation and its molecular mechanisms. To this regard, we conducted *in vitro* and *in vivo* researches to get a comprehensive understanding of EMD.

In this study, we first verified the “stemness” of the expanded colony-forming cells by flow cytometric analysis, multilineage differentiation testing, and immunofluorescence staining. The results demonstrated that largely pure DPSCs were successfully separated from heterogeneous DPCs to guarantee their property for odontogenic differentiation (Figures 1(c) and 1(f)). Then, we observed the cell viability of DPSCs influenced by EMD, and found that various concentrations of EMD did not appear to affect the growth of DPSCs in all tested time points (Figure 2(a)). To determine the optimum concentration of EMD for induction, we assessed ALP activity and calcium deposition of DPSCs. ALP is known as an early indicator for detection of osteoblast and odontoblast differentiation [35, 36], while the presence of mineralized nodules is a crucial marker of osteo/odontoblastic differentiation at a late stage. Our results revealed that 100 *μ*g/mL of EMD enjoyed the best odontoblastic differentiation potential and was therefore selected as the optimal concentration for subsequent analyses. To further confirm the role of EMD during odontoblastic differentiation in DPSCs, the expression of related marker genes, including RUNX2, ALP, BSP, DSPP, DMP1, and OCN, was detected at both mRNA and protein levels. RUNX2 is known as a significant transcription factor that regulates the differentiation of MSCs towards osteoblasts and odontoblasts [37, 38]. The expression of DSPP and DMP1 is specifically associated with odontoblastic differentiation during dentinogenesis [39–42]. Besides, OCN and BSP are both crucial non-collagenous in extracellular matrix of bone and dentin [43, 44]. In the present study, EMD upregulated the mRNA and protein expression levels of these odontoblastic related genes during the early or late stages of mineralization. The results suggested that EMD could enhance the odontoblastic differentiation of DPSCs *in vitro*, which paves the way for its potential role in pulp-dentin regeneration *in vivo*.

In addition to effective signaling factors, appropriate scaffolds could also weigh heavily for the success of tissue engineering, which serve as a three-dimensional (3D) tissue template suitable for competent cells to adhere, proliferate, and differentiate [20]. Since the scaffold materials applied to pulp tissue engineering should be well adapted to the delicate morphology of the root canal system, injectable hydrogels with superior biocompatibility and biodegradability have become the most promising scaffolds for pulp-dentin regeneration [45–47]. VitroGel 3D-RGD used in our experiment is an injectable and adjustable hydrogel system that is modified with a high concentration of RGD cell adhesive peptide to facilitate cell attachments and cell-matrix interactions during the 3D cell culture [48]. Through simple mixing, cells and active molecules can be easily introduced into and evenly distributed in hydrogel that is injected into any irregular-shaped space [49]. This animal origin-free polysaccharide VitroGel maintains its liquid form at room temperature and only starts to polymerize into a gel when mixed with cell culture medium. Notably, the mechanical properties of the hydrogel and rate of hydrogel formation can be adjusted by the concentration of the VitroGel solution and cell culture medium, giving a broad range of elastic modulus of hydrogel strength.

Favorable biocompatibility is the premise for the practical application of scaffolds in biomedical fields. In this study, the ultrastructural feature of VitroGel 3D-RGD and its effect on the growth of DPSCs were observed by means of SEM. The results presented that the VitroGel has a high porosity and homogeneous interconnected 3D network, which allows the hydrogel system to contain large amounts of water to get reliable viscosity while maintaining its shape [48]. Moreover, we observed that VitroGel 3D-RGD could well support the seeding, adhesion, and proliferation of DPSCs, unfolding that the hydrogel has favorable biocompatibility with the cells. The tissue formation following subcutaneous injection of EMD-induced DPSC/VitroGel complexes into nude mice supported our hypothesis that EMD might be the key epithelial signal to promote odontoblastic differentiation. Based on our *in vivo* results, the EMD group formed substantial collagen matrix, osteoid dentin, and mineralized hard tissue compared with the control group. Since both DSPP and OCN are representative components of non-collagenous dentin extracellular matrix, the positive immunohistochemical staining found in the experimental group indicated the presence of odontoblasts in the regenerated tissue. Generally, we proved that EMD enhanced the odontoblastic differentiation of DPSCs *in vivo* with VitroGel 3D-RGD served as an injectable scaffold.

The mechanism by which EMD affects DPSCs differentiation remains unclear. We have further explored the associated signaling pathways controlling the EMD-induced odonto/osteogenic differentiation of DPSCs. It is well documented that cell differentiation is a complex and dynamic process involving multiple growth factors and signaling pathways. One of the vital mechanisms that take part in the regulation of cell proliferation and odonto/osteogenic differentiation is the mitogen-activated protein kinase (MAPK) pathway [50]. MAPK family, a group of serine/threonine protein kinases, weighs heavily for cytodifferentiation by transducing multiple extracellular signals to cellular responses [51]. There are three main MAPK subfamilies in mammalian cells, namely, c-Jun N-terminal kinase (JNK), extracellular signal-regulated kinase (ERK), and p38 kinase (p38) [52]. It has been reported that EMD could enhance production of matrixmetalloproteinase-2 by osteoblasts and promote periodontal regeneration via the p38 MAPK pathway [53]. Besides, EMD promotes cell proliferation and osteoblast differentiation of human MSCs through the ERK 1/2 pathway [54]. Meanwhile, numerous studies have demonstrated the regulatory role of MAPK signaling pathways in osteo/odontoblastic differentiation of DPSCs [50, 51, 55]. Therefore, we assumed that MAPK pathways might be involved in the EMD-enhanced differentiation of DPSCs. In the present study, p-JNK, p-p38 MAPK, and p-ERK were upregulated immediately after EMD treatment, indicating that EMD activated the MAPK pathways in DPSCs. Furthermore, the ALP content, mineralized nodules deposition, and the protein expression levels of odonto/osteogenic-related markers were suppressed after being blocked with specific pathway inhibitors. Thus, blocking the JNK, ERK, and p38 pathways inhibited odontoblastic differentiation of EMD-treated DPSCs. These findings elucidate that EMD may affect the differentiation of DPSCs through triggering MAPK signaling pathways, thereby providing a new insight into the mechanism by which EMD affects DPSCs function. Interestingly, we observed that the inhibitory effect of SP600125 and U0126 was stronger than that of SB203580. That means the JNK and ERK pathways may play a more important role in the EMD-induced differentiation of DPSCs. One limitation of our study is that we only explored the potential mechanisms *in vitro*. Further *in vivo* studies are needed to provide more solid evidence that EMD functions via the MAPK pathways.

## 5. Conclusions

In summary, the present *in vitro* and *in vivo* studies illuminated that EMD could enhance the odontoblastic differentiation of DPSCs via MAPK signaling pathways without interference with DPSCs proliferation. Furthermore, VitroGel 3D-RGD offered a beneficial microenvironment for DPSCs function, displaying its promising application in pulp tissue engineering. Our findings provide a new insight into the mechanism by which EMD affects DPSCs differentiation and the application of EMD in future chairside endodontic treatment. In-depth studies shall be conducted with in situ animal models to further investigate the effect of EMD on pulp-dentin regeneration.

## Figures and Tables

**Table 1:** Primers sequence for real-time RT-PCR.

Gene	Forward	Reverse
RUNX2	GTCTCACTGCCTCTCACTTG	CACACATCTCCTCCCTTCTG
ALP	CCAAGGACGCTGGGAAATCT	TATGCATGAGCTGGTAGGCG
BSP	GCGAAGCAGAAGTGGATGAAA	TGCCTCTGTGCTGTTGGTACTG
DSPP	GAGGTAACACCAGGCACT	TCCCTGCTTCTTCATCTT
DMP1	ACTGTGGAGTGACACCAGAACACA	AGCTGCAAAGTTATCATGCAGATCC
OCN	GCCAGGCAGGTGCGAAGC	GTCAGCCAACTCGTCACAGTCC
GAPDH	GCACCGTCAAGGCTGAGAAC	TGGTGAAGACGCCAGTGGA

## Data Availability

The data used to support the findings of this study are included within the article.
